# Nurse preceptors’ perceptions of benefits, rewards, support, and commitment to the preceptor role in a new preceptorship program

**DOI:** 10.1186/s12909-022-03534-0

**Published:** 2022-06-17

**Authors:** Leila Gholizadeh, Shahla Shahbazi, Sousan Valizadeh, Masoumeh Mohammadzad, Akram Ghahramanian, Masoumeh Shohani

**Affiliations:** 1 Faculty of Health, University of Technology Sydney, Sydney, Australia; 2 Department of Medical-Surgical Nursing, Faculty of Nursing and Midwifery, Tabriz University of Medical Sciences, PO Box 51745347, Tabriz, Iran; 3 Clinical Research Development Unit, Sina Educational, Research and Treatment Center, Tabriz University of Medical Sciences, Tabriz, Iran; 4 Department of Paediatrics, Faculty of Nursing and Midwifery, Tabriz University of Medical Sciences, Tabriz, Iran; 5 Tabriz Children’s Hospital, Tabriz University of Medical Sciences, Tabriz, Iran; 6 Faculty of Allied Medical Sciences, Ilam University of Medical Sciences, Ilam, Iran

**Keywords:** Preceptorship, Preceptor, Nurse, Role commitment, Benefits, Rewards, Support

## Abstract

**Background:**

Nurse preceptorship is a new concept emerging in the Iranian health care system. The purpose of this research was to assess preceptor nurses’ perceived benefits, rewards, support, and commitment to the role in a new nurse preceptorship program in Iran and to examine the relationships between these concepts.

**Methods:**

A descriptive correlational study was employed, and using total population sampling method, 45 preceptor nurses were recruited from a tertiary referral teaching hospital in Iran. Data were collected using the Preceptor’s Perception of Benefits and Rewards Scale, the Preceptor’s Perception of Support Scale, and the Commitment to the Preceptor Role Scale. Descriptive statistics and correlational analysis were used to analyse data.

**Results:**

Preceptors’ commitment to their role was positively and moderately associated with their perceived benefits and rewards (*r* = 0.503, *p* = 0.001) and perceived support (*r* = 0.430, *p* = 0.003). None of the examined demographic and practice variables showed statistically significant association with commitment to the preceptor role.

**Conclusions:**

Commitment to the preceptor role was associated with benefits, rewards and support that preceptor nurses perceive in relation to their role. To optimise the effectiveness of nurse preceptorship programs, benefits, rewards, recognition, and support should be integral to planning of these programs.

## Background

Clinical preceptorship is defined as a formal arrangement between a practicing health professional (the preceptor) and a new graduate or student (the preceptee) within a clinical setting [1]. The preceptorship program has been found as an effective support strategy for newly graduated nurses, helping them gain knowledge, skills, and confidence in their professional role [2]. In some countries, it is mandatory that newly graduated nurses undergo a period of preceptorship prior to achieving a full professional registration [3, 4]. The nurse preceptorship program is increasingly being recognised and implemented in the health care system of Iran [5–8]. However, the evaluation of the effectiveness of this program is limited. In order to successfully implement the program, it is important to understand Iranian preceptor nurses’ experience, their perceived benefits and challenges to help better prepare, reward, and retain them in the role.

Different styles of preceptorship programs are used in healthcare settings [9]. In the hospital where this study was conducted, the preceptorship program was established in line with preparing the workplace to better support new nurses in adapting to their new work environments [10, 11]. In general, the preceptorship program involves the education of newly graduated nurses who enter clinical practice for the first time and orientation of experienced nurses to ensure a smooth transition to a new workplace [12, 13]. The duration of the program is usually between two to 4 weeks. However, the program is extendable depending the preceptee’s needs and abilities. The preceptor is often an experienced nurse who is introduced by the senior nurse manager. Their role is to facilitate the new nurse’s transition to safe nursing practice by orienting them to the new physical environment, ward routines, and norms and values of the workplace. The preceptor prioritises nurses’ educational needs, and helps them to increasingly grow independence and carry out nursing interventions safely [13].

For the successful implementation of the preceptorship program, it is important that the preceptor commit to their role, and for this to occur, there should be adequate benefits, rewards, and support services available [14, 15]. Yet, the relationships between these factors have not been examined in settings where preceptorship program, as a support mechanism, is a new phenomenon. Factors that enhance and sustain preceptorship models as a new phenomenon in care units need a better understanding. Studies that have aimed to evaluate the success of the preceptorship programs have mainly focused on the experience of preceptees and their achievements [16, 17]. More research is needed on nurse preceptors’ perception of their role, as the important partners of the preceptorship program [15]. This study aimed to assess perceived benefits, rewards, support, and commitments to the role of preceptors involved in preceptorship of newly graduated nurses in Iran, and test this hypothesis that preceptor nurses’ commitment to their role is associated with their perceived benefits, rewards, and support concerning their role.

### Conceptual framework

The conceptual framework of this study is based on Kanter’s theory of structural empowerment. The theory suggests that access to information, resources, and support services as well as opportunities to learn and develop are key factors in empowering employees in an organisation. Staffs should receive appreciation and rewards for their contribution to organisational goals, and have opportunities for personal and career developments [18]. This study assumed that preceptor nurses who perceive benefits and opportunities in their preceptorship role, that there are adequate support services available, and their contributions are recognised and valued, they would be better committed to their preceptor role.

## Methods

### Research design

This study used an exploratory, descriptive correlational design to examine the experience of preceptor nurses being engaged in a new nurse preceptorship program.

### The study setting

The setting of this study was Tabriz Children’s Hospital, the largest children’s hospital in the northwest of Iran. At the time of the study, the hospital was implementing a nurse preceptorship program, which to the researchers’ knowledge; it was the first program of this kind being formally implemented in the health care system of Iran.

In this hospital, the unit manger nominated the preceptor nurse based on some criteria including having a permanent employment position, at least 1 year of clinical experience in the relevant ward, not planning to go on a long-term leave, willingness to work with new nurses, having a positive attitude towards nursing profession, exhibiting good communication skills, being a positive role model as a nurse, and interested in teaching and learning. The preceptorship program was offered both to newly graduated nurses with no previous work experience, experienced nurses who were are newly employed by the hospital, and to nurses who were transferred from another ward of the hospital to a new ward with a different specialty and routines.

The preceptorship period lasted between two to 4 weeks, during which the new nurse worked under supervision of a preceptor nurse. The duration of the preceptorship program was extendable to maximum 1 month depending on the learning needs of the new nurse, the complexity of the work in the ward, and based on the decision of the preceptor nurse and the unit manager. However, due to nurse shortage, it was often difficult for the hospital to extend the preceptorship period. There were at least two preceptors in each hospital ward to firstly prevent burnout related to long term preceptor role and to substitute the primary preceptor when they were on leave for any reason.

In the context of this study, the educational supervisor of the hospital approved the suitability of the nominated preceptor nurse for the program and coordinated the preceptorship program. The educational supervisor monitored, maintained, and evaluated the quality of the preceptorship program and liaised between the director of nursing, preceptor nurses, preceptee nurses, and other members involved in the preceptorship program, including unit mangers and nurses on the floor. The educational supervisor monitored the improvement of preceptee nurses and participated in identifying their learning needs and performance problems.

### Participants and data collection

Using total population sampling method, nursing preceptors were recruited from different wards of the participating hospital. To be included in the study, participants needed to be currently working as the preceptor for new nurses in the hospital and have completed the preceptorship program with at least two new nurses previously. Nurses who had been preceptors to nursing students only were excluded from the study. The list of preceptors in each ward was obtained from the director of nursing. The researcher met with each preceptor individually, explained the study objectives, screened them against the study inclusion and exclusion criteria, and invited all eligible preceptors to the study. There were 50 preceptors working in the hospital, of whom 46 were eligible for inclusion in the study, and 45 preceptors accepted the invitation (response rate 97.82%). Data collection was completed in March 2016.

### Definition of terms

The definitions of the key concepts by Dibert and Goldenberg (1995) were adopted in this study [19]. Benefits and rewards were defined as positive outcomes associated with the preceptorship experience, and measured by the Preceptor’s Perception of Benefits and Rewards (PPBR) scale [19]. Support was defined as factors that enhance and facilitate the preceptor role, and measured by the Preceptor’s Perception of Support (PPS) scale [19]. Finally, commitment to the role was defined as a dedication to a particular course of action, and measured by the Commitment to the Preceptor Role (CPR) scale [19].

### Instruments

The study questionnaire was composed of 38 items, developed by Dibert and Goldenberg in 1995 to measure preceptors’ perception of benefits and rewards associated with the preceptor role, perceived support, and commitment to the role [19]. The questionnaire possesses three sub-scales of Preceptor’s Perception of Benefits and Rewards containing 14 items (the PPBR), Preceptor’s Perception of Support containing 17 items (the PPS), and Commitment to the Preceptor Role containing 10 items (the CPR). The items are rated on a 6-point Likert scale, with responses ranging from 1 ‘strongly disagree’ to 6 ‘strongly agree [19] Higher scores indicate greater perceived benefits, rewards, support, and commitment to the role. Five items, including items 17, 20, 21, 27, and 30 on the PBS scale, and four items, including items 34, 37, 38, and 40 on the CPR scale, are negatively scored [20]. The scores for these items were reversed when analyzing the responses. The validity and reliability of the questionnaire have been established internationally [21, 22]. In the original study, Cronbach’s Alpha coefficients for the three scales of the PPBR, the PPS, and the CPR were reported as 0.91, 0.86, and 0.87, respectively [19].

Permission was obtained from the developers to translate and use the questionnaire for the current study (Dolly Goldenberg, Personal Communication, Oct 24, 2013). The Persian translation of the scale was carried out using the forward-backward translation procedure recommended by Guillemin et al. [23]. It was ensured that the translated versions of the items accurately conveyed the meaning of the items employed in the original scale. Before using the questionnaire, three nursing academics and seven clinical nurses reviewed the Persian versions of the scale and confirmed the clarity of the items. In current study, all the sub-scales demonstrated good internal consistency, with Cronbach’s alphas of 0.88, 0.75, and 0.71 for the CPR, the PPBR, and the PPS sub-scales, respectively. Demographic and professional characteristics were collected including age, gender, nursing qualification, years of work experience as a registered nurse, employment status, number of preceptorships completed, years of work experience as the preceptor, and preceptor training.

### Ethical considerations

The ethics committee of Tabriz University of Medical Sciences (TBZMED.REC.1394.773) approved the study. Participants were provided with both verbal and written information about the research, and their voluntary participation, anonymity, and confidentiality were considered during the study process. Signed consent was obtained from each participant.

### Data analysis

The Statistical Package for Social Sciences (SPSS-PC, version 14, SPSS Inc., Chicago, IL, USA) was used for data entry and analysis. Descriptive statistics, including means and standard deviations, were used to summarise demographic and practice data and data on the sub-scales. The relationships between the CPR, the PPBR, and the PPS scores were determined using Spearman’s correlation coefficient. A *p*-value < 0.05 was considered statistically significant.

## Results

Participants were all female and with age range between 26 to 46 years (35.86 ± 4.62). All participants were employed on a full-time basis, and they had a bachelor’s degree in nursing, except for two participants who had postgraduate degrees. Their work experience as registered nurses ranged from 5 to 20 years (12.5 years ±4.12), and they had precepted at least ten new nurses previously, with their preceptorship experience ranging from 1 to 5 years (3.15 ± 1.46). None of participants had received any form of formal preceptorship training at the time of the study (Table 1).

**Table 1:** Demographic and professional characteristics of the preceptors (*N* = 45)

Variable	N (%)	Mean	Standard deviation	Range
**Age**		35.86	4.62	20.00
20-29	3 (7.0%)			
30-39	29 (67.4%)			
≥ 40	11 (26.6%)			
**Highest earned degree**				
BSN	43 (95.6%)			
MSN	2 (4.4%)			
**Years of nursing experience**		12.02	4.12	15.00
**Years of preceptor experience**		3.15	1.46	4.00
**Number of preceptor experiences with new nurses**		4.68	.72	2.00
4-7 new nurses	6 (14.6%)			
8-10 new nurses	1 (2.4%)			
> 10 new nurses	34 (82.9%)			
**Overall preceptorship experience**				
Positive	34 (81.0%)			
Negative	0 (0.0%)			
Both Positive and Negative	1 (2.4%)			
**Neither positive nor negative**	7 (16.7%)			

### Preceptor’s perception of benefits and rewards (the PPBR)

The mean of PPBR scores was high (5.33 ± 0.62), meaning that the participants perceived high benefits and rewards concerning their preceptor role. On this scale, Item 2, *assisting new staff nurses and nursing students to integrate into the nursing unit*, was the most highly rated item (5.73 ± 0.54), followed by Item 9, *sharing my knowledge with new nurses and nursing students* (5.73 ± 0.50), Item 1, *teach new nurses and nursing students* (5.67 ± 0.60) and Item 4, *keep current and remain stimulated in my profession* (5.49 ± 0.84). The lowest rated item was Item 5, *influence change in my nursing unit* (4.64 ± 1.50), followed by Item 14, *improve my chances for promotion/advancement within this organisation* (4.82 ± 1.39), and Item 15, *improve my organisational skills* (5.04 ± 1.24) (Table 2).

**Table 2:** The mean, standard deviation, and range of item level scores and total scores of the preceptor’s perception of benefits and rewards scale (*n* = 45)

No	Item	Mean	Standard deviation	Range
1	Teach new staff nurses and nursing students.	5.67	.60	2.00
2	Assist new nurses and nursing students to integrate into the nursing unit.	5.73	.54	2.00
3	Increase my own professional knowledge base.	5.38	1.13	5.00
4	Keep current and remain stimulated in my profession.	5.49	.84	4.00
5	Influence change in my nursing unit.	5.16	1.04	4.00
6	Gain personal satisfaction from the role.	5.40	.86	4.00
7	Be recognized as a role model.	5.29	.94	4.00
8	Improve my teaching skills.	5.62	.58	2.00
9	Share my knowledge with new nurses and nursing students.	5.73	.50	2.00
10	Learn from nurses and nursing students.	5.24	1.00	4.00
11	Contribute to my profession.	5.47	.87	4.00
12	Increase my involvement within my workplace.	4.64	1.59	5.00
13	Improve my organizational skills.	5.04	1.24	4.00
14	Improve my chances for promotion/advancement within my workplace.	4.82	1.39	5.00
**Total**		**5.33**	**.62**	**2.07**

To sum up, having opportunities to contribute to knowledge development and skill acquisition of new nurses, and keeping up to date and stimulated in nursing profession were perceived as benefits of being a preceptor nurse. However, they did not perceive much benefit in the role in terms of having ability to influence change in the workplace, increasing their chance of job promotion, or gaining organisational skills.

### Preceptor’s perceptions of support (the PPS)

The mean total PPS scores were low (3.37 ± 0.62), meaning that the participants perceived low support in relation to their preceptor role. On this scale, Item 15, *I feel I have adequate preparation for my role as preceptor*, was the highest-rated item (5.29 ± 0.55), followed by Item 23, *nursing coordinators are available to help me develop in my role as a preceptor* (4.70 ± 1.24), and Item 24, *nursing educators are available to help me develop in my role as preceptor* (4.49 ± 1.24). The lowest rated item was Item 25, *there are adequate opportunities for me to share information with other preceptors* (2.91 ± 1.39), followed by Item 19, *my workload is appropriate when I function as a preceptor* (2.29 ± 1.48), and Item 17, *the nursing staff do not understand the goal of the preceptor program* (2.70 ± 1.25) (Table 3).

**Table 3:** The mean, standard deviation, and range of item level scores and total scores of the perception of support scale (*N* = 45)

No	Item	Mean	Standard deviation	Range
15	I feel I have had adequate preparation for my role as a preceptor.	5.29	.55	2.00
16	My goals as a preceptor are clearly defined.	4.20	1.55	5.00
17	The nursing staff do not understand the goal of the preceptor program.(R)	2.70	1.30	5.00
18	My co-workers on the nursing unit are supportive of preceptor program.	3.64	1.49	5.00
19	My workload is appropriate when I function as a preceptor.	2.29	1.49	5.00
20	I do not have sufficient time to provide patient care while I function as a preceptor.(R)	2.07	1.05	4.00
21	I feel I function as a preceptor too often.(R)	3.18	1.25	5.00
22	I feel the nurse manager is committed to the success of the preceptor program.	4.27	1.35	4.00
23	I feel my head nurse is committed to the success of the preceptor program.	4.70	1.24	4.00
24	Educational supervisor is available to help me develop in my role as a preceptor.	4.49	1.24	5.00
25	There are adequate opportunities for me to share information with other preceptors.	2.91	1.39	5.00
26	Educational supervisor provides support by helping me to identify a new nurse^’^s performance problems.	3.49	1.67	5.00
27	Educational supervisor spends too little time with the new nurse.(R)	3.16	1.49	5.00
28	The guidelines clearly outline the responsibilities of the nursing educational supervisor in relation to my preceptor role.	3.41	1.42	5.00
29	The nursing faculty member provides support by helping me to identify a student^’^s performance problems.	1.97	1.37	4.00
30	The nursing faculty member spends too little time with the nursing student.(R)	2.87	1.52	5.00
31	The guidelines clearly outline the responsibilities of the nursing faculty member in relation to my preceptor role.	2.18	1.34	4.00
**Total**		**3.37**	**0.62**	**2.44**

The support that preceptor nurses perceived in relation to their role included having adequate preparation for their role and the availability of support from nursing coordinators and nurse educators. However, participants did not perceive much support from other nurses or preceptors or in relation to their workload.

### Commitment to the preceptor role (the CPR)

The participants ranked their commitment to the preceptor role as moderate (4.49 ± 0.75). They gave lowest scores to Item 34, *I feel very little loyalty to the preceptor program* (5.33 ± 0.13). The scores on this item were reversed, and therefore, the results indicated that participants perceived themselves as highly loyal to the program. They also rated highly Item 32, *I am willing to put in a great deal of effort beyond what is normally expected in order to help the preceptor be successful* (5.29 ± 0.89), and Item 41, *being a preceptor, inspires me to perform my very best* (5.22 ± 1.26). The lowest rankings on this scale were related to Item 37, *it would take very little change in my present circumstances to cause me to stop being a preceptor* (2.82 ± 1.81), and in Item 38, *there is not too much to be gained by continuing to be a preceptor* (2.55 ± 1.60) (Table 4).

**Table 4:** The mean, standard deviation, and range of item level scores and total scores of the preceptor’s perception of role commitment (*n* = 45)

No	Item	Mean	Standard deviation	Range
32	I am willing to put in a great deal of effort beyond what is normally expected in order to help the new nurses be successful.	5.29	.895	4.00
33	I am enthusiastic about the preceptor program when I talk to my nursing colleagues.	4.40	1.71	5.00
34	I feel very little loyalty to the preceptor program.(R)	5.33	1.128	5.00
35	I find that my values and the values of the preceptor program are very similar.	4.86	1.04	4.00
36	I am proud to tell others that I am a preceptor.	4.51	1.70	5.00
37	It would take very little change in my present circumstances to cause me to stop being a preceptor.(R)	2.82	1.81	5.00
38	There is not too much to be gained by continuing to be a preceptor.(R)	2.55	1.60	5.00
39	I really care about the fate of the preceptor program in this hospital/community.	4.82	1.23	5.00
40	Deciding to be a preceptor was a definite mistake on my part.(R)	5.16	1.12	4.00
41	Being a preceptor really inspires me to perform my very best.	5.22	1.26	5.00
**Total**		**4.49**	**0.75**	**3.20**

To sum up, the preceptor nurses in this study rated their commitment to the role as moderate. They perceived themselves as highly loyal to the program and were willing to put in a great deal of effort to the role. They perceived that the preceptor role inspired them to perform their best. However, they were not confident about staying in the role or did not see too much to gain from continuing to be in the preceptor role.

### Correlations between preceptors’ perceptions of benefits and rewards and support and their commitment to the preceptor role

Spearman’s correlation coefficient was used to examine relationships between the preceptor’s perceptions of benefits, rewards, and support and their commitment to the role of preceptorship (Table 5). There was a statistically significant positive and moderate correlation between perception of benefits and rewards (the PPBR) and commitment to the role (the CPR) (*r* = 0.503, *p* < 0.001). There was also a statistically significant positive and moderate correlation between the preceptors’ perception of support for the preceptor role (the PPS) and commitment to the role (the CPR) (*r* = 0.430, *p* = 0.003).

**Table 5:** Associations between the commitment to the role and preceptors’ perceived benefits and rewards and preceptors’ perceived support scores

	The CPR	The PPBR	The PPS
The CPR	–	*r* = 0.430 *p* = 0.003**	*r* = 0.503 *p* < 0.001**
The PPBR	*r* = 0.329 *p* = 0.027*	–	–
The PPS	–	–	–

*r* = Spearman Rank Order Coefficient

*Correlation is significant at the 0.05 level (2-tailed)

**Correlation is significant at the 0.01 level (2-tailed)

None of the demographic and practice variables was significantly correlated with the PPBR, the PPS, or the CPR.

## Discussion

The success of the preceptorship program depends very much on the commitment of preceptors to their role [15]. In this study, the preceptors rated their commitment to the preceptor role as moderate, which is consistent with previous international research [15, 19, 21, 22, 24]. These findings indicate the need for a better understanding of the preceptors’ viewpoints, experience, and perceived barriers and facilitators to help meet their needs and enhance their commitment to the role.

Preceptor nurses in this study perceived overall high benefits and rewards from their preceptor role. They found the opportunity to teach, share knowledge, and assist novice nurses in developing into professional care providers as rewarding and satisfying. In addition, they believed the preceptor role helped them keep up to date and stimulated in their profession. However, they did not believe that being in the preceptor role increased their ability to influence change in their workplace, or increased their chance for job promotion or acquisition of organisational skills. Previous research indicates that preceptors can be valuable to the organisation in implementing their new policies and strategies [25]. Seemingly, the participating hospital was missing out on opportunities to improve the quality of its services and apply changes through the involvement of the preceptors, which could also in turn give the preceptors a sense of mattering and making a difference in the workplace. Perception of benefits and rewards has been found to be positively associated with commitment to the role of preceptorship both in this study and previous research [15, 19, 22]. To encourage preceptors’ commitment to their role, health care organisations should ensure that there are sufficient benefits and rewards in relation to the preceptorship role; for example, having an increased chance for job promotion or a reduced or flexible workload.

It is also likely that preceptors do not sufficiently identify the potential benefits associated with the role, such as opportunities to keep up to date with new knowledge and advances in the field through studying or learning from new nurses, or improving their organisation skills, such as teaching skills, interpersonal skills, team working, and leadership. When recruiting nurses for the preceptor role, setting clear goals, defining expectations, and explaining potential benefits and challenges may help preceptors develop a realistic expectation about the role. This can also help preceptors see the positive aspects of their preceptor role rather than just the challenges [26], an appreciation that may increase preceptor nurses’ commitment to their role.

In line with international research [19, 21, 22, 24, 27], this study found a statistically significant positive correlation between preceptors’ perceived support and commitment to the role. However, the overall level of perceived support was low in this study, which is inconsistent with previous reports (Table 6) [19, 21, 22, 24]. Unlike the preceptorship programs for nursing students, in which preceptors are largely supported by the faculty, in new nurse preceptorship programs, preceptors do not receive this kind of support [28, 29]. The preceptor nurses in this study perceived that they were adequately prepared for their role as a preceptor and were sufficiently supported by nurse coordinators and nurse educators about their role. In the participating hospital, at the time of conducting this study, there was no formal and structured preparation for preceptors, such as a preceptorship course or training [5]. However, the hospital’s educational supervisor coordinated the preceptorship program and acted as the preceptor for preceptors, providing support and education as needed. This finding is consistent with Whitehead et al.’s (2016) study, suggesting that nurse educators can play an important role in supporting the preceptors and should be considered as part of the preceptorship management support structure [30].

**Table 6:** Comparison of the results of the current study with the past research

Scale	Current Study (2016)	Chang et al. (2013)	Hyrkäs & Shoemaker (2007)	Usher et al. (1999)	Dibert & Goldenberg (1995)
Preceptors’ Perception of Benefits and Rewards	5.33	5.04	4.93	4.88	4.56
Preceptors’ Perception of Support	3.37	4.36	3.78	3.64	4.03
Commitment to the Preceptor Role	4.49	4.74	4.81	Not reported	Not reported

The preceptors believed that they lacked opportunities to share their experiences with other preceptors and receive their support. Peer discussions could enrich knowledge and provide preceptor nurses with mental support and a sense of connectedness and support. It is believed that learning is a social, cultural, and emotional process rather than a solely cognitive process. Preceptors should have opportunities to socialise with other preceptors and members of interdisciplinary teams to obtain knowledge and skills which could be beneficial to their preceptor role [31].

In addition, the preceptors in this study perceived little support from their co-workers in relation to the program. As part of facilitating the implementation of a preceptorship program, nurses on the floor should have a clear understanding of the program and the role of the preceptor nurse, to help enhance their collaboration with and support of the program. Findings of other studies also confirm that preceptor nurses often do not attain adequate support and collaboration from co-workers [5, 32, 33]. A supportive environment and co-workers can contribute to a better preceptorship experience [34], and the preceptorship program should be built on a collaborative and supportive culture to ensure that the program is a positive and rewarding experience for all [34]. Even nurses who are not selected to serve as preceptors could benefit from a new colleague who is well-prepared to deliver safe and quality patient care. This understanding is likely to increase their support of the program, for example, through accepting additional responsibilities to free the preceptor’s time or engaging with the new nurse to make them feel welcomed and supported in the new workplace [35].

The preceptors in the current study stated that their workload was too heavy to allow them to function efficiently as a preceptor. Consistent with other studies, they were nominated by unit managers on the basis of their merits and experience [36], however, the program coordinators discounted the workload involved in undertaking the preceptor role on top of patient care responsibilities. In another similar study, preceptors described their experience as ‘living with moral distress’: they felt responsible for the professional development of new nurses but could not fulfill their responsibilities as they wished [13]. A reasonable workload allocation should be considered for preceptor nurses to reduce the risk of compromising one role to another and help optimise the outcomes of the preceptorship program [37, 38].

Similar to findings of previous studies [19, 39, 40], this study results provide support to Kanter’s theory of structural empowerment. That is preceptor nurse who perceived benefits and opportunities in their preceptorship role, and believed that they were adequate support services available in relation to their role, they were more likely to report a higher commitment to their role.

### Study limitations

The findings of this study added to our understanding of the nurse preceptorship program. More specially, the research focused on the experience of preceptor nurses, in terms of their perceived benefits, rewards, and support regarding the preceptor role and examined the relationships between these concepts and preceptors’ commitment to their role. Nevertheless, the study has some limitations to consider. This is a single-center study with small sample size, although the setting was a tertiary referral hospital, and preceptors were recruited from different wards with a high response rate (97.82%) to the study invitations. All participating preceptors were female; therefore, the findings of this study may not reflect the experience of preceptors working in other hospitals or of male preceptors. The study focused on perceptions of benefits, rewards, support, and commitment to the preceptor role of preceptors engaged in a nurse preceptorship program, therefore, the findings of this study may not be generalised to preceptors who provide preceptorship to nursing students. It is interesting to study how the relationship between the preceptor nurse and the new nurse would affect the preceptors’ commitment to their role particularly that they are supposed to work as colleagues in the same organisation in the near future. Qualitative or mixed method studies are needed to help fully explore different factors that affect preceptor nurses’ experience and their commitment to the role.

## Conclusions

To our knowledge, this is the first study that investigated preceptor nurses’ perception of benefits, rewards, support, and their commitment to the role in a new nurse preceptorship program in Iran. Preceptors in this study reported moderate commitment to their preceptor role. Commitment to the preceptor role was significantly, although moderately, associated with perceived benefits, rewards, and support. Overall, the preceptors perceived a high level of benefits and rewards about their preceptor role; however, they rated lowly on the support they received, particularly in relation to their workload, and support from co-workers and other preceptors.

The success of a preceptorship program depends partially on the commitment of the preceptor to the role. The commitment to the role may be improved through providing preceptors with sufficient benefits and rewards, resources and support. A qualitative exploration of preceptor nurses’ experience would allow a better understanding of multiple and complex factors that affect commitment and loyalty to the preceptor role. This learning can help with designing interventions to optimise the effectiveness of preceptorship programs.

## Data Availability

The datasets generated and analyzed during the current study are not publicly available due to the confidentiality of the participants but are available from the corresponding author on reasonable request.

## Abbreviations

PPBR: Preceptor’s Perception of Benefits and Rewards
PPS: Preceptor’s Perceptions of Support
CPR: Commitment to the Preceptor Role
